# Concurrent vaccination against equine influenza and equine herpesvirus – a practical approach

**DOI:** 10.1111/irv.12396

**Published:** 2016-07-02

**Authors:** Sarah Gildea, Maria Jose Sanchez Higgins, Gillian Johnson, Cathal Walsh, Ann Cullinane

**Affiliations:** ^1^ Virology UnitThe Irish Equine CentreNaasCo. KildareIreland; ^2^ HawkfieldNewbridgeCo. KildareIreland; ^3^ Department of Mathematics and StatisticsUniversity of LimerickLimerickIreland

**Keywords:** Concurrent, herpesvirus, influenza, racehorse, vaccination

## Abstract

**Background:**

There is a lack of information concerning concurrent administration of vaccines against equine influenza virus (EIV) and equine herpesvirus 1 and 4 (EHV‐1/4).

**Objectives:**

The primary objective of this study was to determine the impact of the concurrent use of EIV and EHV‐1/4 vaccines in Thoroughbred racehorses on their humoral immune response to EIV.

**Methods:**

This study was carried out on a population of 30 horses using an inactivated whole‐virus EIV vaccine and an inactivated EHV‐1/4 vaccine. Horses were randomly allocated to vaccination group A or B. Horses in group A were vaccinated against EIV and EHV‐1/4 2 weeks apart. Horses in group B were vaccinated against EIV and EHV‐1/4 on the same day. Whole‐blood samples were collected on the day of vaccination and 2 weeks and 6 weeks post‐vaccination. Antibody levels against EIV and EHV‐1/4 were measured using the single radial haemolysis and serum neutralisation test, respectively.

**Results:**

The pattern of EIV antibody response post‐vaccination was similar for both groups. Highest EIV antibody levels were recorded 2 weeks post‐vaccination, and a significant decrease in antibody level was observed 4 weeks later. Horses in group B demonstrated a significantly higher EIV antibody response post‐vaccination. Overall, there was no significant difference in EHV‐1/4 antibody response between the two groups post‐vaccination.

**Conclusion:**

In this study, concurrent vaccination against EIV and EHV‐1/4 increased the response to EIV and did not compromise the humoral immune response to EHV‐1/4.

## Introduction

Epidemiological investigations of acute respiratory disease have confirmed that equine influenza virus (EIV) and equine herpesvirus 1 and 4 (EHV‐1/4) are important causes of both clinical and subclinical infection among young horses in racing yards and stud farms, and those returning from equestrian events.^1^, ^2^, ^3^, ^4^, ^5^ Disease and suboptimal performance following infection with these viruses can result in significant financial loss. Equine viral diseases are primarily controlled by vaccination, and in the absence of multivalent vaccines, vaccines against different viruses may be given concurrently to simplify management and to minimise veterinary expense. It is not known, however, to what extent concurrent administration may compromise the humoral response to the individual vaccine preparations. It has been demonstrated repeatedly in vaccine trials and in the field that antibodies against EIV haemagglutinin as measured by single radial haemolysis (SRH) correlate with protection against influenza provided the vaccine strains are closely related to those circulating in the field.^6^, ^7^, ^8^ However, unlike EIV there are no definitive immune correlates of protection to assess vaccine efficacy against EHV‐1/4. Nevertheless, in several vaccination studies serum‐neutralising (SN) antibodies have correlated with protection against some clinical signs and reduced duration of virus shedding.^9^, ^10^, ^11^ The objective of this study was to evaluate virus‐specific antibody titres induced following concurrent and consecutive vaccination against EIV and EHV‐1/4 using the inactivated whole‐virus EIV vaccine Duvaxyn IE Plus and the inactivated bivalent EHV‐1/4 vaccine Duvaxyn EHV‐1,4.

## Material and methods

### Horses

This study was carried out on a population of 30 Thoroughbred 2‐year‐olds in a racing yard. The sample size was based on the available number of new arrivals and represented a large intake for a flat training yard in Ireland. It was decided to carry out the study in a single yard as it minimised potentially confounding factors and the racehorse trainer wished to determine which vaccination regime was of most benefit to the horses in his care.

### Vaccines

The inactivated whole‐virus EIV vaccine Duvaxyn IE Plus and inactivated bivalent EHV‐1/4 vaccine Duvaxyn EHV‐1,4 were purchased commercially by the trainer's veterinary surgeon. Duvaxyn IE Plus contained inactivated A/eq/1/Prague/56 (H7N7), the prototype H7N7 virus; A/eq/Suffolk/89 (H3N8), a representative of the European lineage; and A/eq/Newmarket/1/93 (H3N8), a representative of the American lineage. Duvaxyn EHV‐1,4 contained inactivated EHV‐1 strain 438/77 and inactivated EHV‐4 strain 405/76. Both vaccines were adjuvanted with carbopol.

### Vaccinations

All horses had at least completed their primary EIV vaccination course prior to entering the training yard. Irrespective of this study, these horses were scheduled for vaccination as part of the preventive health measures routinely implemented for new arrivals. They were previously unvaccinated against EHV; however, the majority were seropositive for EHV‐4 on initial sampling. Horses were randomly allocated to vaccination group A (15 horses) or B (15 horses). Horses in group A were vaccinated against EIV (Duvaxyn IE Plus) and EHV‐1/4 (Duvaxyn EHV‐1,4) 2 weeks apart. Horses in group B were vaccinated against EIV and EHV‐1/4 on the same day. Vaccination was performed by the resident veterinary surgeon.

### Collection of samples

Whole‐blood samples were collected by the trainer's resident veterinary surgeon from the horses on the day of vaccination, followed by 2 weeks and 6 weeks post‐vaccination. The collection of blood samples to monitor viral antibody titres and more specifically response to vaccination is an integral part of the routine veterinary care in this training yard. Samples were submitted to the laboratory following collection, and serum was stored at −20°C until testing.

### Serology

All samples were tested for antibodies against EIV of the H3N8 subtype. Antibodies against A/eq/Donegal/09 (H3N8) and A/eq/Meath/07 (H3N8) representatives of the currently circulating Florida sublineage Clade 1 and Florida sublineage Clade 2 viruses, respectively, were measured using the single radial haemolysis (SRH) test as previously described.^12^ A significant rise in antibody titre was defined as an increase in the mean H3N8 SRH level of 25 mm^2^ or 50%, whichever is smaller between the acute and convalescent serum samples.^6^

Antibodies against Irish field isolates of EHV‐1 (strain 146375) and EHV‐4 (strain 122324) were measured using the serum neutralisation test (SNT) for 27 of the 30 horses in accordance with standard procedure.^13^ There was insufficient sample available to test three horses, one in group A and two in group B. End‐point virus neutralisation antibody titres were calculated by determining the reciprocal of the highest serum dilution that protected 100% of the cell monolayer (rabbit kidney‐13 cells for EHV‐1 and primary equine embryonic lung cells for EHV‐4) from virus destruction. Seroconversion was defined as a fourfold or greater rise in SNT antibody titre. The laboratory investigator was blinded to the vaccination schedule allocation of individual horses.

### Statistical analysis

All statistical analysis was carried out on the open‐source package r version 3.1.1. Data were analysed using the Wilcoxon and independent *t*‐tests. The Wilcoxon rank test was used to examine the SNT results as changes are recorded on the basis of fold increase which was not normally distributed. Given that the SRH values over time were approximately normally distributed, a paired *t*‐test was used for longitudinal analysis, that is to compare the values at different times post‐vaccination. Similarly, to compare SRH values between the two groups, that is cross‐sectional analysis, a two‐sample *t*‐test was used. The area under the curve (AUC) as described by Heldens *et al*.^14^ was calculated by the trapezoidal rule and used as the metric for the repeated‐measures analysis of EIV antibody levels.

## Results

**Figure 1:**
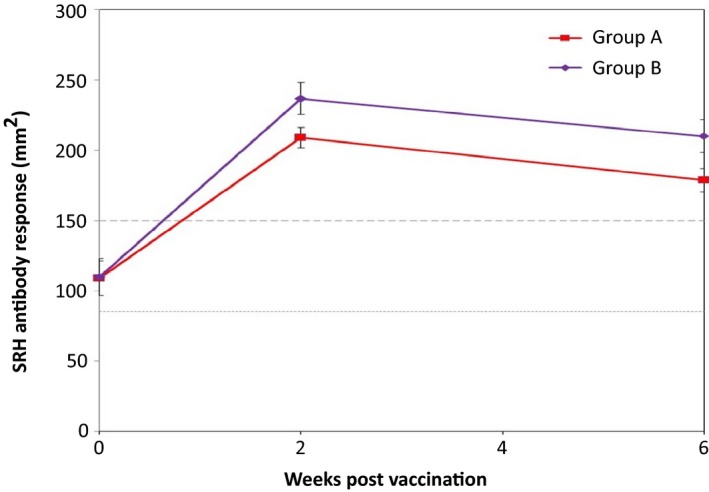
Mean H3N8 single radial haemolysis (SRH) antibody response measured in the weeks following booster vaccination. Broken lines = SRH antibody levels 85 mm^2^ and 150 mm^2^ correlating with clinical and virological protection, respectively. Error bars represent standard error of the mean.

No adverse clinical reactions post‐vaccination were observed in any of the horses. There was no significant difference (*P* = 0·96) in mean H3N8 EIV antibody levels between horses in group A (109 ± 12·3 mm^2^ SE) and horses in group B (110 ± 13·1 mm^2^ SE) prior to booster vaccination. Fourteen of the horses in group A (93%) and 14 of the horses in group B (93%) seroconverted to EIV following vaccination. The two horses that did not seroconvert had a mean H3N8 antibody level of 178 mm^2^ and 185 mm^2^, respectively, prior to booster vaccination. The pattern of EIV antibody response post‐vaccination was similar for both groups (Figure 1). Highest antibody levels were recorded 2 weeks post‐vaccination (mean H3N8 antibody level: group A = 209·0 ± 7·2 mm^2^ SE; group B = 237 ± 11·3 mm^2^ SE). There was a significant decrease in H3N8 antibody levels between 2 and 6 weeks post‐booster vaccination (*P* < 0·001) when the mean H3N8 antibody level for group A and group B was 179 ± 8·2 mm^2^ SE and 210 ± 11·7 mm^2^ SE, respectively. Horses in group B demonstrated a significantly higher antibody response 2 weeks (*P* = 0·049) and 6 weeks (*P* = 0·038) post‐booster vaccination compared to horses in group A. The AUC of the SRH titres was also calculated, and a significant difference between the groups was established (*P* = 0·037).

**Figure 2:**
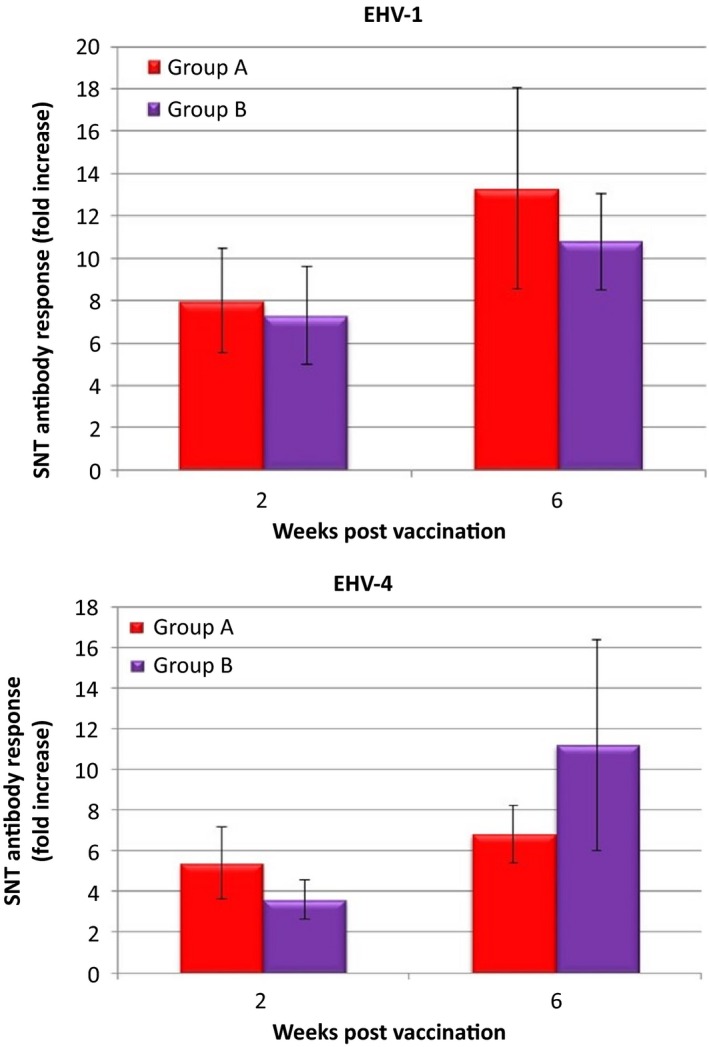
(A) Mean fold increase in equine herpesvirus‐1 (EHV‐1) antibody level 2 weeks and 6 weeks post‐vaccination. Error bars represent standard error of the mean.(B) Mean fold increase in EHV‐4 antibody level 2 weeks and 6 weeks post‐vaccination. Error bars represent standard error of the mean.

**Figure 3:**
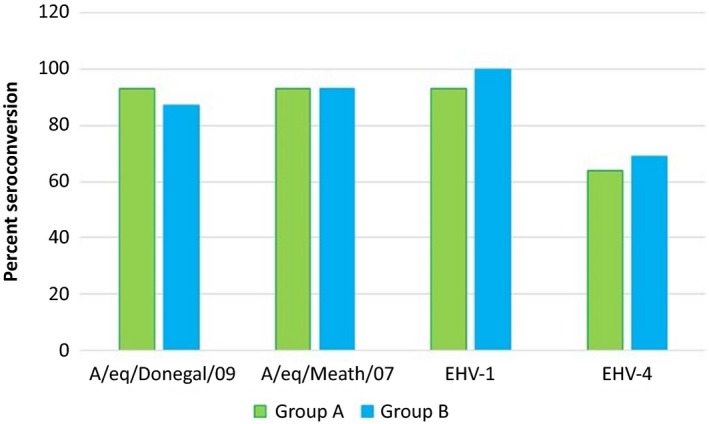
The percentage of horses in groups A and B that seroconverted to equine influenza virus (EIV), equine herpesvirus‐1 (EHV‐1) and equine herpesvirus‐4 (EHV‐4) following vaccination.

All horses were seronegative for EHV‐1 prior to vaccination, but only one horse in each group was seronegative for EHV‐4. Thirteen of the 14 horses (93%) in group A and all 13 horses (100%) in group B that were tested by SNT seroconverted to EHV‐1 following vaccination. Nine of the 14 (64%) horses in group A and nine of the 13 (69%) horses in group B seroconverted to EHV‐4 following vaccination. The pattern of EHV‐1 antibody response was similar for both groups in that highest antibody levels were recorded 6 weeks post‐vaccination. Highest antibody levels against EHV‐4 were recorded 6 weeks and 2 weeks post‐vaccination for groups A and B, respectively. Fold increase in EHV‐1/4 antibody level 2 weeks and 6 weeks post‐vaccination is illustrated in Figure 2A,B. Overall, there was no significant difference in EHV‐1/4 antibody response post‐vaccination between the two groups. The response to both vaccines is summarised in Figure 3.

## Discussion and conclusion

This study is the first to examine the serological response of Thoroughbred horses in training to concurrent and consecutive vaccination against EIV and EHV‐1/4. Prior to booster vaccination, there was no significant difference in EIV antibody levels between the two groups and their mean H3N8 antibody levels were similar to that previously observed in Irish racing yards.^12^, ^15^ Ninety‐three per cent of horses in this study seroconverted to EIV post‐booster vaccination. The two horses that did not seroconvert both had SRH antibody level ≥150 mm^2^ at the time of booster vaccination; that is, they would have been considered virologically protected against homologous virus.^6^, ^7^, ^8^ A significant correlation between pre‐existing SRH antibody levels and response to vaccination has previously been established in horses in training.^15^ In this study, there was a significant difference in EIV antibody response between the two groups; that is, the horses that received both vaccines concurrently mounted a greater antibody response. In a comparative vaccine study carried out in young Thoroughbred horses, it was proposed that the superior antibody response elicited by Duvaxyn IE‐T Plus was due to the inclusion of two adjuvants, that is carbomer and aluminium hydroxide.^16^ The findings of this study suggest that the effect of administering two carbopol‐adjuvanted vaccines concurrently may be of benefit in increasing the EIV antibody response compared to when the EIV vaccine is administered alone; however, further investigations in a larger population of horses are required.

All horses in this study were EHV‐1‐seronegative on initial sampling, and with the exception of one, all seroconverted following vaccination. Twenty‐five of the 27 horses (93%) were EHV‐4‐seropositive prior to vaccination, and 18 of the 27 (67%) seroconverted. Of the nine horses that did not seroconvert, eight (89%) were EHV‐4‐seropositive on initial sampling. Early epizootiologic studies in the United States indicated that approximately 85% of foals experience EHV respiratory infections in the 6‐ to 8‐month period after weaning and the majority of outbreaks are caused by EHV‐4.^5^, ^17^ This is the first study to examine the serological response to EHV‐1/4 vaccination in young Thoroughbred racehorses. Previous studies in naive non‐Thoroughbred weanlings indicated that SNT antibody titres were barely detectable 2 weeks post‐first vaccination using the same vaccine.^11^ The absence of seronegative status and the strong anamnestic response observed against both EHV subtypes to one vaccine dose in this study suggest that EHV infection is prevalent among young Thoroughbred horses in Ireland. Overall, no significant difference in EHV‐1/4 antibody response was observed between horses in the two groups.

In conclusion, the results of this study suggest that the two vaccines investigated are compatible and that concurrent vaccination against EIV and EHV‐1/4 does not compromise the humoral immune response against either vaccine. In this study, there was no evidence of interference due to antigenic competition. In fact, it appears that a higher antibody response may be elicited against EIV when the influenza vaccine is administered at the same time as the EHV vaccine. These results are timely in that there is no longer a combined EIV and EHV‐1/4 vaccine available in Ireland or the UK. The practice of concurrent vaccination against EIV and EHV‐1/4 appears to be efficacious and may be advantageous to the owner/trainer in terms of affording greater protection against EIV and reducing the cost associated with vaccination. It also may be of benefit to the animal in improving health and minimising veterinary intervention. However, this study was restricted to a limited number of horses, and further investigation is warranted to determine whether the results are applicable to the general population. The only EHV‐1/4 vaccine available in Ireland was used in the study, but there are several equine influenza vaccines on the market, and it would be useful to determine whether the same results are achieved with different products and how they are affected by previous vaccination. Similarly, it would be beneficial to monitor the duration of the antibody response to equine influenza post‐vaccination to determine whether the higher antibody response observed after concurrent vaccination persists and is of clinical significance to the horses.

## Source of funding

The laboratory work which was carried out at the Irish Equine Centre was funded by the Department of Agriculture, Food and the Marine.

## References

[1] Gildea S , Arkins S , Cullinane A . Management and environmental factors involved in equine influenza outbreaks in Ireland 2007‐2010. Equine Vet J 2011; 43:608–617. 21496094 10.1111/j.2042-3306.2010.00333.x

[2] Gildea S , Fitzpatrick DA , Cullinane A . Epidemiological and virological investigations of equine influenza outbreaks in Ireland (2010‐2012). Influenza Other Respi Viruses 2013; 4:61–72. 10.1111/irv.12192 PMC5655889 24224821

[3] Woodward AL , Rash AS , Blinman D *et al* Development of a surveillance scheme for equine influenza in the UK and characterisation of viruses isolated in Europe, Dubai and the USA from 2010‐2012. Vet Microbiol 2014; 169:113–127. 24480583 10.1016/j.vetmic.2013.11.039

[4] Gilkerson JR , Whalley JM , Drummer HE , Studdert MJ , Love DN . Epidemiological studies of equine herpesvirus 1 (EHV‐1) in Thoroughbred foals: a review of studies conducted in the Hunter Valley of New South Wales between 1995 and 1997. Vet Microbiol 1999; 68:15–25. 10501158 10.1016/s0378-1135(99)00057-7

[5] Doll ER , Bryans JT . Immunization of young horses against viral rhinopneumonitis. Cornell Vet 1963; 53:24–41. 14028463

[6] Newton JR , Townsend HG , Wood JL , Sinclair R , Hannant D , Mumford JA . Immunity to equine influenza: relationship of vaccine‐induced antibody in young Thoroughbred racehorses to protection against field infection with influenza A/equine‐2 viruses (H3N8). Equine Vet J 2000; 32:65–74. 10661388 10.2746/042516400777612116

[7] Mumford JA , Wilson H , Hannant D , Jessett DM . Antigenicity and immunogenicity of equine influenza vaccines containing a Carbomer adjuvant. Epidemiol Infect 1994; 112:421–437. 8150017 10.1017/s0950268800057848 PMC2271453

[8] Mumford JA . Biology, epidemiology and vaccinology of equine influenza. Proceedings of the International Symposium, Budapest 2001; 10–11:7–12.

[9] Goehring LS , Wagner B , Bigbie R *et al* Control of EHV‐1 viremia and nasal shedding by commercial vaccines. Vaccine 2010; 28:5203–5211. 20538091 10.1016/j.vaccine.2010.05.065

[10] Goodman LB , Wagner B , Flaminio MJ *et al* Comparison of the efficacy of inactivated combination and modified‐live virus vaccines against challenge infection with neuropathogenic equine herpesvirus type 1 (EHV‐1). Vaccine 2006; 24:3636–3645. 16513225 10.1016/j.vaccine.2006.01.062

[11] Heldens JG , Hannant D , Cullinane AA *et al* Clinical and virological evaluation of the efficacy of an inactivated EHV1 and EHV4 whole virus vaccine (Duvaxyn EHV1,4). Vaccination/challenge experiments in foals and pregnant mares. Vaccine 2001; 19:4307–4317. 11457558 10.1016/s0264-410x(01)00131-1

[12] Gildea S , Arkins S , Cullinane A . A comparative antibody study of the potential susceptibility of Thoroughbred and non‐Thoroughbred horse populations in Ireland to equine influenza virus. Influenza Other Respi Viruses 2010; 4:363–372. 10.1111/j.1750-2659.2010.00163.x PMC4634612 20958930

[13] OIE 2014 Manual of diagnostic tests and vaccines for terrestrial animals. Equine Rhinopneumonitis Chapter 2.5.9; 894–903.

[14] Heldens JG , Weststrate MW , van den Hoven R . Area under the curve calculations as a tool to compare the efficacy of equine influenza vaccines‐a retrospective analysis of three independent field trials. J Immunol Methods 2002; 264:11–17. 12191504 10.1016/s0022-1759(01)00571-3

[15] Gildea S , Arkins S , Walsh C , Cullinane A . A comparison of antibody responses to commercial equine influenza vaccines following annual booster vaccination of National Hunt horses ‐ a randomised blind study. Vaccine 2011; 29:3917–3922. 21419776 10.1016/j.vaccine.2011.03.003

[16] Gildea S , Quinlivan M , Murphy BA , Cullinane A . Humoral response and antiviral cytokine expression following vaccination of thoroughbred weanlings – a blinded comparison of commercially available vaccines. Vaccine 2013; 31:5216–5222. 24021309 10.1016/j.vaccine.2013.08.083

[17] Allen GP , Bryans JT . Molecular epizootiology, pathogenesis, and prophylaxis of equine herpesvirus‐1 infections. Prog Vet Micro Immunol 1986; 2:78–144. 2856183

